# Correction: Minimising the payload solvent exposed hydrophobic surface area optimises the antibody–drug conjugate properties

**DOI:** 10.1039/d4md90009j

**Published:** 2024-02-22

**Authors:** Adrian D. Hobson, Haizhong Zhu, Wei Qiu, Russell A. Judge, Kenton Longenecker

**Affiliations:** a AbbVie Bioresearch Center 381 Plantation Street Worcester Massachusetts 01605 USA adrian.hobson@abbvie.com; b AbbVie Inc. 1 North Waukegan Road North Chicago IL 60064 USA

## Abstract

Correction for ‘Minimising the payload solvent exposed hydrophobic surface area optimises the antibody–drug conjugate properties’ by Adrian D. Hobson *et al.*, *RSC Med. Chem.*, 2024, https://doi.org/10.1039/d3md00540b.

The authors regret that there is an error in the structure of compound **3** in Fig. 2 of their manuscript. The 3-aminobenzyl group should be in the *para* position instead of the *meta* position as is shown. The corrected scheme is shown below.

**Fig. 2:**
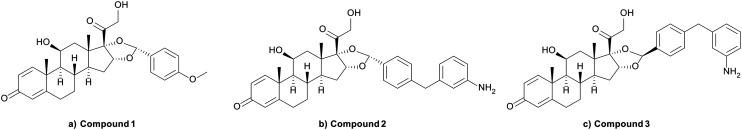
GRM compounds a) mono aryl anisole compound **1**, b) biaryl aniline with (*R*)-acetal compound **2**, c) biaryl aniline with (*S*)-acetal compound **3**.

The Royal Society of Chemistry apologises for these errors and any consequent inconvenience to authors and readers.

## Supplementary Material

